# EDNRB inhibits the growth and migration of prostate cancer cells by activating the cGMP-PKG pathway

**DOI:** 10.1515/med-2023-0875

**Published:** 2024-01-04

**Authors:** Xun Li, Bide Liu, Shuheng Wang, Qiang Dong, Jiuzhi Li

**Affiliations:** Department of Urology, People s Hospital of Xinjiang Uygur Autonomous Region, Urumqi, Xinjiang Uygur Autonomous Region, China; Department of Urology, People s Hospital of Xinjiang Uygur Autonomous Region, No. 91, Tianchi Road, Tianshan District, Urumqi, Xinjiang Uygur Autonomous Region, China

**Keywords:** prostate cancer, B-type endothelin receptor, cGMP-PKG, GEO, cancer

## Abstract

Prostate cancer (PCa) represents a substantial global health concern and a prominent contributor to male cancer-related mortality. The aim of this study is to explore the role of B-type endothelin receptor (EDNRB) in PCa and evaluate its therapeutic potential. The investigation employed predictive methodologies encompassing data acquisition from the GEO and TCGA databases, gene screening, enrichment analysis, *in vitro* experiments involving PCR, Western blotting, wound healing, and Transwell assays, as well as animal experiments. Analysis revealed a significant downregulation of EDNRB expression in PCa cells. Overexpression of EDNRB demonstrated inhibitory effects on tumor cell growth, migration, and invasion, likely mediated through activation of the cGMP-Protein Kinase G pathway. *In vivo* experiments further confirmed the tumor-suppressive properties of EDNRB overexpression. These findings underscore the prospect of EDNRB as a therapeutic target for PCa, offering novel avenues for PCa treatment strategies.

## 1 Introduction

Prostate cancer (PCa) is a significant global health concern and a leading cause of cancer-related mortality. Despite extensive research efforts, the prognosis for advanced PCa remains worrisome due to limited sensitive biomarkers for early detection and effective treatment. Therefore, there is an urgent need to explore the molecular mechanisms underlying PCa and identify novel biomarkers for early diagnosis and treatment [1,2].

Protein Kinase G (PKG) serves as a major cGMP second messenger receptor, modulating intracellular signaling pathways involved in cell differentiation, platelet activation, memory formation, and vasodilation. PKG1 exhibits tumor suppressor properties, while cGMP-dependent PKG2 has been reported to inhibit the proliferation of certain cancer cells, including glioma cells, and promote apoptosis in breast cancer cells. Activation of the cGMP-PKG pathway has demonstrated inhibitory effects on proliferation, migration, and invasion of PCa cells [3,4].

Additionally, our research focuses on B-type endothelin receptor (EDNRB). The EDNRB gene, located on chromosome 13 q22 with GenBank ID 1910, spans approximately 24 kb and comprises 7 exons and 6 introns. Its promoter region contains a CpG island that may undergo hypermethylation-induced inactivation. EDNRB is a member of the G protein-coupled receptor family, transmitting extracellular signals through binding to the ligand endothelin. During embryonic development, EDNRB plays a crucial role in the migration and differentiation of neural crest cells, which is relevant to Hirschsprung’s disease. Studies have revealed associations between hypermethylation of the EDNRB gene promoter and various tumors, often accompanied by decreased EDNRB gene expression. Notably, EDNRB has been found to inhibit proliferation and migration in lung cancer cell line H1299, exhibit lower expression levels in hepatocellular carcinoma compared to adjacent tissues, and its hypermethylation reduces expression in oral squamous cell carcinoma lesions. Conversely, re-expression of EDNRB can alleviate cancer-induced pain [5,6,7].

Despite these findings, the role of EDNRB in PCa and its underlying mechanisms require further elucidation. The advancements in chip technology and high-throughput sequencing have significantly contributed to cancer research, enabling the identification of biomarkers for cancer diagnosis, treatment, and prognosis. Integrating bioinformatics methods [8], coupled with data mining across multiple databases, offers a robust and accurate analysis by leveraging a larger pool of clinical samples. These innovative bioinformatics techniques hold substantial potential for advancing cancer biomarker research [9]. Thus, our study aims to leverage these approaches, combining bioinformatics analysis and *in vitro* experimental verification, to unravel the role of EDNRB in PCa and provide new insights for diagnosis and treatment strategies.

## 2 Methods

### 2.1 Data acquisition and differential gene screening

The expression profile of PCa-related data was retrieved from the GEO database (https://www.ncbi.nlm.nih.gov/geo/). The study included the microarray dataset (GSE69223), consisting of 15 PCa patients and 30 matched malignant and non-malignant prostate tissue samples. Differential mRNA expression was analyzed using the R packages Limma and ggord, with adjusted *P* values (<0.05) and log2 fold change thresholds (>1 or <−1) applied. Expression heatmaps were generated using the R package pheatmap, and Venn diagrams were created using ggVennDiagram. The differentially expressed genes obtained were compared with those identified in the TCGA database for PCa.

### 2.2 KEGG enrichment analysis of DEGs

Enrichr, a comprehensive tool for genome enrichment analysis (available at http://amp.pharm.mssm.edu/Enrichr/), was utilized to conduct biological pathway analysis on differentially expressed genes using the KEGG pathway database. The top ten pathways with significant enrichment were visualized and downloaded directly from the online platform.

### 2.3 Gene expression analysis

The Gene Expression Profiling Interactive Analysis (GEPIA) database, available at http://gepia.cancer-pku.cn/detail.php, encompasses RNA-sequencing data from 8,587 normal samples and 9,736 tumor samples obtained from the Genotype-Tissue Expression Dataset Project and TCGA Expression data. In this study, we utilized the GEPIA database to examine the expression of the EDNRB gene in PCa samples compared to normal controls.

### 2.4 Cell culture

The human PCa cell lines PC3, DU145, LNCaP, and 22Rv2, as well as the normal prostate epithelial cell line RWPE-1, were procured from the Cell Resource Center, Peking Union Medical College. The specified cell culture mediums for each cell line were as follows: PC3 and DU145 were cultured in Dulbecco’s Modified Eagle’s Medium (DMEM) with 10% fetal bovine serum (FBS); 22RV2 and LNCap cells were cultured in Roswell Park Memorial Institute (RPMI) medium with 10% FBS. The normal RWPE cell lines were grown in keratinocyte-free serum medium supplemented with bovine pituitary extract and recombinant epidermal growth factor. All cells were maintained in a humidified incubator at 37°C with 5% CO_2_. Experiments were conducted within the first five passages after culture initiation. Cells were cultured in T-175 flasks with 30 ml of their respective media until reaching 70% confluency for subsequent harvesting or passaging.

For the analysis of EDNRB mRNA and protein expression, the cells were grouped as RWPE-1, LNCap, 22RV2, DU145, and PC-3. When studying the effect of EDNRB, DU145 and PC-3 cells were utilized, and the groups consisted of a control group, a transfection vector group, and an EDNRB overexpression group. For investigating the effect of EDNRB on signaling pathways, the groups were divided into a control group, an EDNRB overexpression group, and an EDNRB overexpression combined with (D)-DT-2 (PKG inhibitor) group. Finally, the animal experiments included a vector transfection group and an EDNRB overexpression group.

### 2.5 Animals

The animal experiments conducted in this study were granted ethical approval by the ethics committee of People’s Hospital of Xinjiang Uygur Autonomous Region. A total of 40 male BALB/c nude mice, aged 4–6 weeks and weighing 20–25 g, were used in the study. The mice were housed in a specific pathogen-free environment that included individually ventilated cages and an isolator module. To initiate the study, the treated PCa cells (1 × 10^6^) were injected into the axilla of the mice. Subsequently, PC-3 cells, PC-3 cells transfected with a vector, or PC-3 cells overexpressing EDNRB were subcutaneously injected into the BALB/c nude mice. The BALB/c nude mice injected with PC-3 cells served as negative controls. Tumor volumes were regularly monitored and measured for up to 24 days, while tumor weights were assessed upon euthanizing the mice.

### 2.6 Cell transfection

Plasmid vectors were constructed by Hanbio (Shanghai, China), and the pcDNA3.1 vector was used to insert the human EDNRB cDNA. Transfection was carried out using Lipofectamine 2,000 (Invitrogen, USA) following the manufacturer’s protocol. Prior to transduction, MSCs were seeded at a density of 1 × 10^5^ cells per well in 12-well plates. The medium (500 μl/well) was then replaced with fresh serum-free DMEM medium. For each transduction, a viral vector (approximately 2.5 μl) was pre-mixed with 20 μl of HiTransGP transfection reagent (Genechem) and added to each well to achieve a multiplicity of infection of 50. After 12 h of transfection, the medium was replaced with DMEM containing 10% FBS. Following a 48 h incubation period, cells were observed under a fluorescent microscope to identify GFP + cells. Successfully transfected cells were selected using puromycin.

### 2.7 Polymerase chain reaction

Total RNA was extracted from cells using an Agbio RNA extraction kit (Hunan, China). Subsequently, cDNA synthesis was performed using a kit from Agbio (Hunan, China) with 1 μg of total RNA as the template. Quantitative PCR was conducted on a 7,500 ABI system using 1× SYBR reagent (Applied Biosystems) to measure the transcript level of EDNRB. The primer sequences used were as follows: EDNRB (forward: GGCTCCTACTATCCTGGTTCTG, reverse: CAAGGCAAGCATAACACCAGTGC) and β-actin (forward: GGCTCCTACTATCCTGGTTCTG, reverse: CAAGGCAAGCATAACACCAGTGC).

### 2.8 Western blot (WB)

The protein expression of EDNRB was evaluated using WB analysis. Briefly, 30 μg of total protein was loaded onto SDS-polyacrylamide gels (4–15%; Bio-Rad) and subsequently transferred to PVDF membranes (Bio-Rad). The membranes were then incubated overnight at 4°C with a 5% blocking solution (2.5 g nonfat dry milk in Tris buffer containing 0.1% Tween, TBST). Following this, the membranes were probed with rabbit polyclonal antibodies against EDNRB (Proteintech, Cat No. 20964-1-AP, 1:1,000), PKG1 (Proteintech, Cat No. 21646-1-AP, 1:500), PKG2 (Proteintech, Cat No. 55138-1-AP, 1:500), and β-actin (Proteintech, Cat No. 81115-1-RR, 1:500) for 1 h at room temperature. Subsequently, the membranes were washed six times with TBST (three 15 min washes followed by three 5 min washes) and then incubated with a goat anti-rabbit secondary antibody conjugated to alkaline phosphatase (Proteintech, Cat No. SA00001-2, 1:3,000) or a goat anti-mouse secondary antibody conjugated to alkaline phosphatase (Proteintech, Cat No. SA00001-1, 1:3,000) for 1 h at room temperature. After washing with TBST, the proteins were visualized using an ECL detection reagent (Lablead, Beijing, China). The expression of β-actin served as an internal control.

### 2.9 Cell counting kit (CCK)-8 assay

The number of viable cells was determined using the CCK-8 assay (Solarbio, Beijing, China). A cell suspension was inoculated into a 96-well plate at a density of 2 × 10^3^ cells per well and pre-incubated for 24 h after transfection. Treated and untreated cells were cultured accordingly. Subsequently, 10 µl of CCK-8 solution was added to each well, and the cells were incubated at 37°C for 2 h. The absorbance at 450 nm was measured using a TECAN Infinite M200 multimode microplate reader (Tecan, Mechelen, Belgium).

### 2.10 Cell migration and invasion assays

The constructed vector and EDNRB vector group cells were seeded at a density of 1 × 10^5^ cells per well in 24-well culture plates and incubated at 37°C with 5% CO_2_ for 24 h. After removing the media, the cell surface was gently scratched using a 10 μl pipette tip. The cells were washed twice with PBS and then 1 ml of RPMI 1640 medium was added. Scratch images were captured at 0 and 24 h. The experiment was performed in triplicate and repeated three times. The distance of cell migration into the wound area during this period was measured.

Cell invasiveness was assessed using Transwell chambers with an 8 µm pore size (Corning, USA). Cells (5–7 × 10^4^ cells per chamber) were seeded into the upper chamber in 200 µl of serum-free medium. The lower chamber was filled with 600 μl of DMEM or RPMI 1640 supplemented with 10% FBS. Matrigel (Corning, USA) was added to the upper chamber as in the cell invasion assay. After 48 h of culture, cells were fixed with 4% paraformaldehyde for 30 min, followed by staining with 0.1% crystal violet (Beyotime, Shanghai, China) for another 30 min. Non-invaded cells on the upper surface of the membrane were removed using a damp cotton swab. Stained cells that migrated or invaded the membrane were photographed under an inverted microscope at 10× magnification, and counts were performed in five randomly selected areas.

### 2.11 Colony formation assay

Cells were seeded at a density of 5 × 10² cells per well in triplicate on six-well plates. The cells were cultured for 12 days with medium changes performed every 4 days. At the end of the 12 days period, colonies were fixed using ice-cold 100% methanol for 20 min, followed by staining with 0.1% crystal violet at room temperature for 20 min. Subsequently, the plates were washed for 10 min in a room temperature water bath and air dried overnight. Quantification of colony formation was performed by calculating the percentage of area covered per well.

### 2.12 Ki-67 staining

The mouse tumor tissue sections were subjected to deparaffinization and rehydration using an ethanol gradient. Subsequently, these sections were treated with an anti-Ki-67 antibody (Proteintech, Cat No: 27309-1-AP, 1:3,000). Following PBS washes, the paraffin sections were exposed to a secondary antibody, specifically goat anti-rabbit. Hematoxylin was employed as a counterstain, and the expression of Ki-67 was examined under an optical microscope. For analysis, ten fields per section were captured.

### 2.13 Statistical analysis

All experiments were conducted in triplicate, and the results are presented as the mean value and standard deviation. Prior to employing GraphPad Prism 8 (GraphPad, La Jolla, CA) for data analysis and graphical representation, we initially conducted normality and variance homogeneity tests to ascertain whether the data distribution and variance fulfilled the assumptions necessary for one-way analysis of variance (ANOVA) and the *t*-test hypothesis. Subsequently, ANOVA or *t*-test was conducted to determine if there existed a significant disparity in the mean values among the groups. In cases where a significant difference was observed, Tukey’s post hoc test was subsequently utilized to accurately identify the specific groups between which the differences occurred. For all tests, a significance level of *P* < 0.05 was employed, and any result with a *P* value lower than 0.05 was considered statistically significant.


**Ethics approval:** Ethical approval was obtained from the Ethics Committee of People’s Hospital of Xinjiang Uygur Autonomous Region.

## 3 Results

### 3.1 Differentially expressed gene profile in PCa

**Figure 1:**
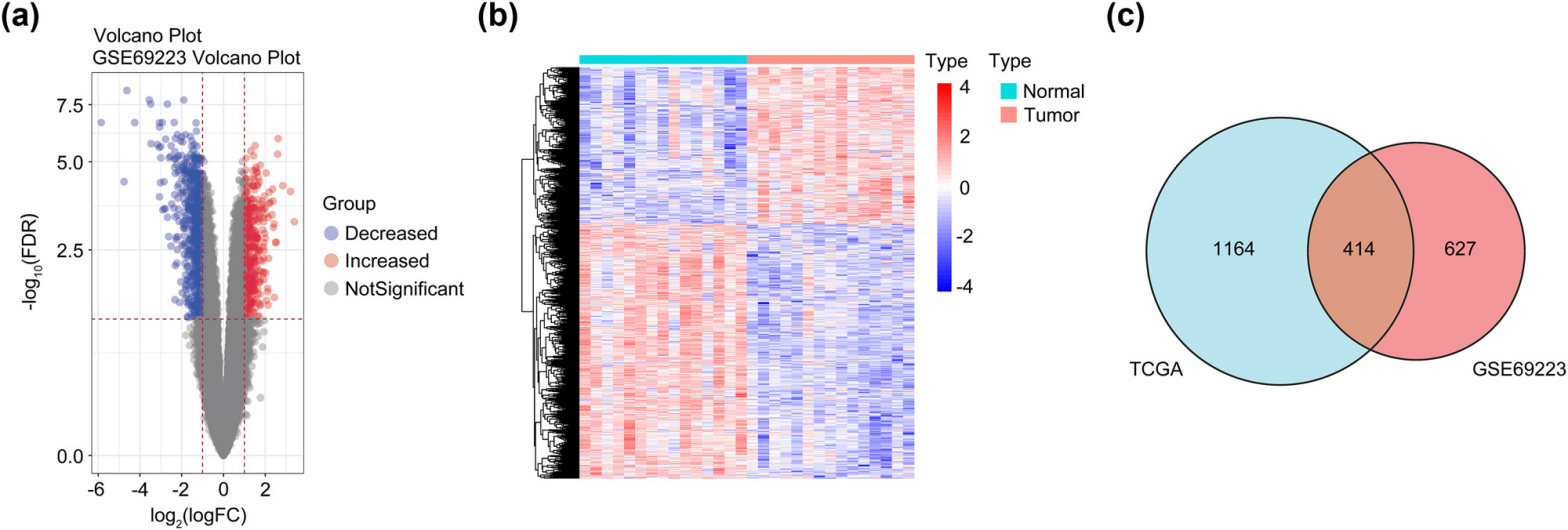
The differential gene results of GSE69223 expression profile mining and the intersection results with TCGA. (a) Volcano map of differential genes. (b) Heat map. (c) The result of the intersection of the differential genes of the two databases is displayed in a Venn diagram.

**Figure 2:**
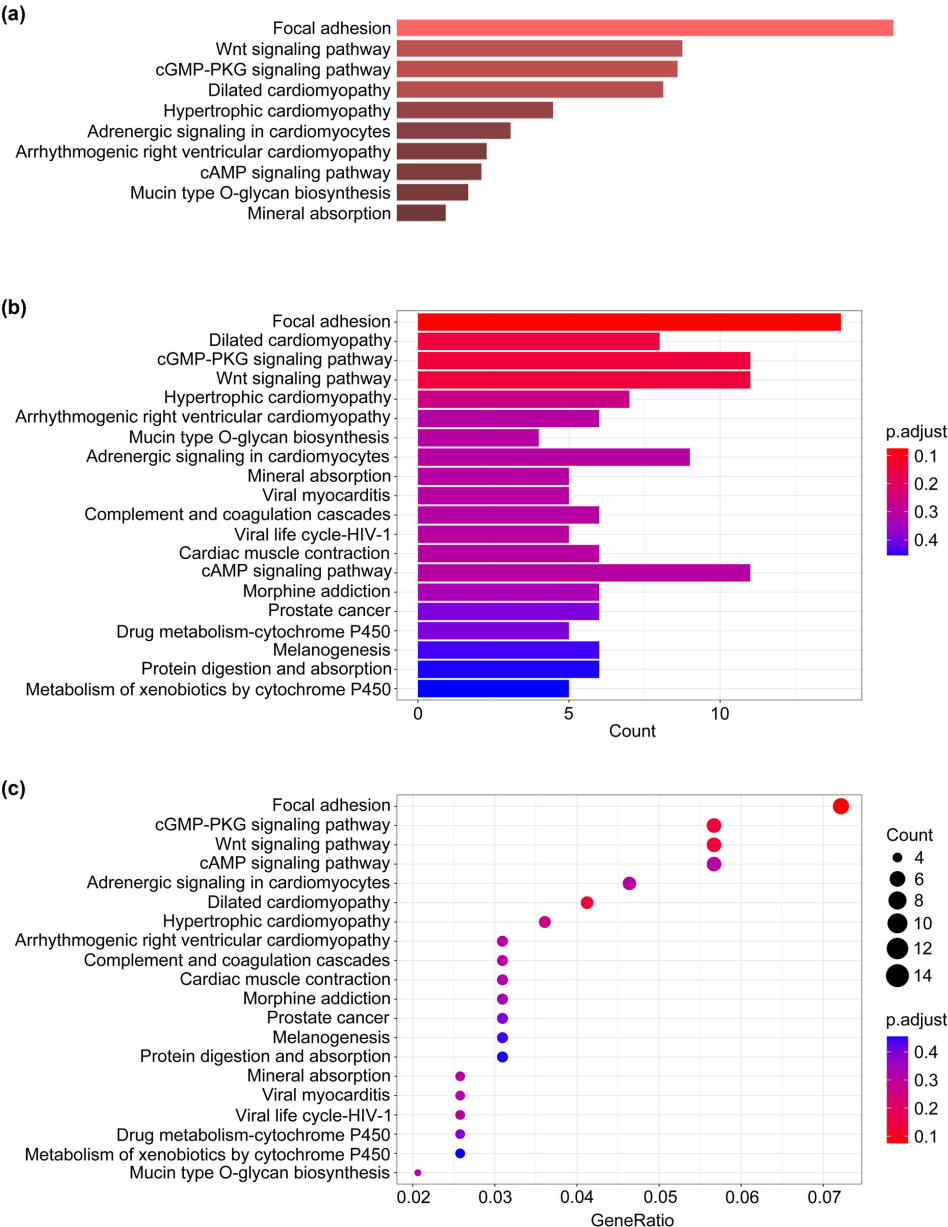
The result of enriching differential genes through Enrichr’s KEGG. (a) Enrichr results, showing the top ten enriched pathways. (b) KEGG enriched pathway status bar graph. (c) Bubble graph.

**Figure 3:**
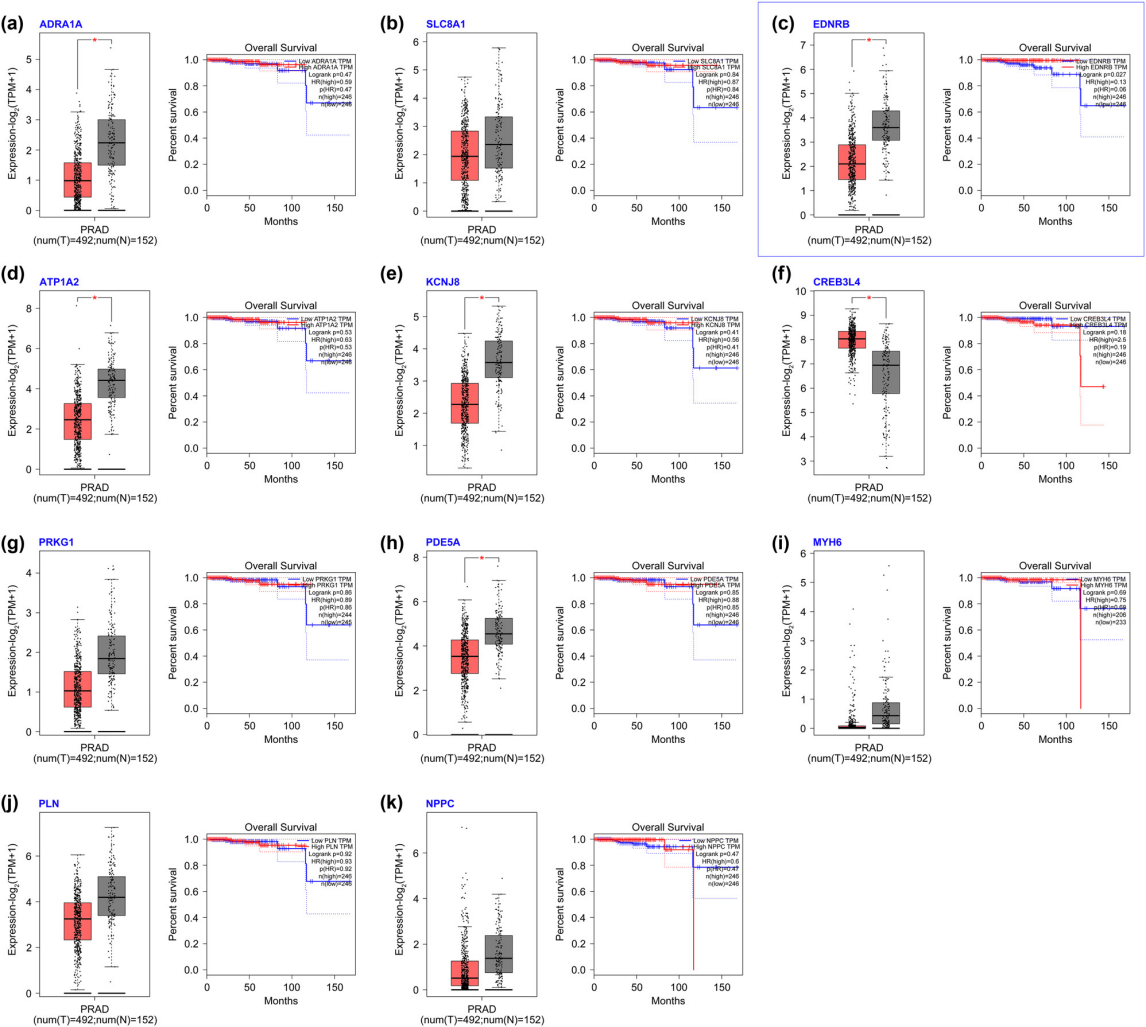
Survival analysis results of genes in the cGMP-PKG pathway and PCa prognosis: (a) ADRA1A. (b) SLC8A1. (c) EDNRB1. (d) ATP1A2. (e) KCNJ8. (f) CREB3L4. (g) PRKG1. (h) PDE5A. (i) MYH6. (j) PLN. (k) NPPC.

From the GSE69223 profile from GEO, 1,041 differential genes were identified (Figure 1a), and the volcano map and heat map were created using R language (Figure 1b). After comparing these genes with differentially expressed genes of PCa in the TCGA database, 414 intersection genes were found (Figure 1c). KEGG pathway enrichment analysis revealed cGMP-PKG and Wnt as the primarily enriched pathway (Figure 2a–c). Given cGMP-PKG pathway’s reported role in inhibiting PCa cells’ proliferation, migration, and invasion, it was chosen for further study. The gene expression enriched in this signaling pathway is listed in Table 1. Prognostic impacts of the gene expressions in this pathway were examined using the GEPIA database, revealing the EDNRB gene’s correlation with prostate patient survival (Figure 3a–i).

**Table 1:** Genes enriched in the cGMP-PKG signaling pathway

Pathway	Gene	Annotation
cGMP–PKG	ADRA1A	Adrenoceptor alpha 1A, involved in smooth muscle contraction and cell proliferation regulation
	SLC8A1	Solute carrier family 8 member A1, crucial for calcium transport and cellular homeostasis
	EDNRB	Endothelin Receptor Type-B, plays a role in vasoconstriction and cell proliferation, a focus in PCa
	ATP1A2	ATPase Na^+^/K^+^ transporting subunit alpha 2, maintains electrochemical gradients for muscle and nerve function
	KCNJ8	Potassium channel involved in regulating vascular tone and potassium ion transport
	CREB3L4	Transcription factor influencing cell growth and survival
	PRKG1	Protein kinase cGMP-dependent 1, phosphorylates proteins, involved in smooth muscle relaxation
	PDE5A	Phosphodiesterase 5A, regulates cGMP breakdown and signaling intensity
	MYH6	Myosin Heavy Chain 6, part of the cardiac muscle contractile apparatus
	PLN	Phospholamban, regulates the cardiac muscle cell calcium pump
	NPPC	Natriuretic Peptide C, involved in blood pressure reduction and may influence PCa progression

Note: cGMP-PKG, cGMP-dependent protein kinase (PKG) signaling pathway; ADRA1A, adrenoceptor alpha 1A; SLC8A1, solute carrier family 8 member A1; EDNRB, Endothelin Receptor Type B; ATP1A2, ATPase Na^+^/K^+^ transporting subunit alpha 2; KCNJ8, Potassium inwardly rectifying channel subfamily J member 8; CREB3L4, Cyclic AMP-responsive element-binding protein 3-like protein 4; PRKG1, PRKG1 protein kinase cGMP-dependent 1; PDE5A, Phosphodiesterase 5A; MYH6, Myosin Heavy Chain 6; PLN, Phospholamban; NPPC, Natriuretic Peptide C.

### 3.2 Expression analysis of EDNRB gene in PCa

**Figure 4:**
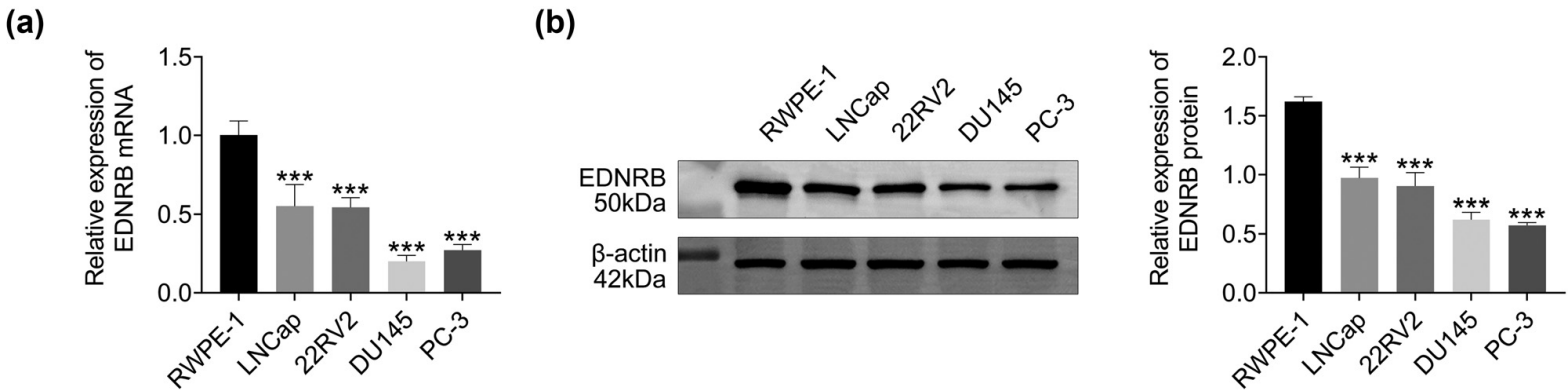
EDNRB1 expression in RWPE-1 human normal prostate epithelial cells, PCa cell lines LNCap, DU145, PC-3, and 22RV2. (a) PCR result and (b) WB result. *N* = 3, ***p < 0.001 vs the RWPE-1 cell group.

To validate the findings from the previous analysis, the role of the EDNRB gene in PCa was investigated using PCR and WB techniques. The results of PCR and WB experiments demonstrated consistent findings, indicating that the mRNA and protein expression levels of the EDNRB gene were significantly reduced in LNCap, DU145, PC-3, and 22RV2 cells compared to RWPE-1 cells (Figure 4a and b). These preliminary results suggest that the EDNRB gene is expressed at lower levels in PCa cells.

### 3.3 EDNRB gene inhibits PCa cell growth

**Figure 5:**
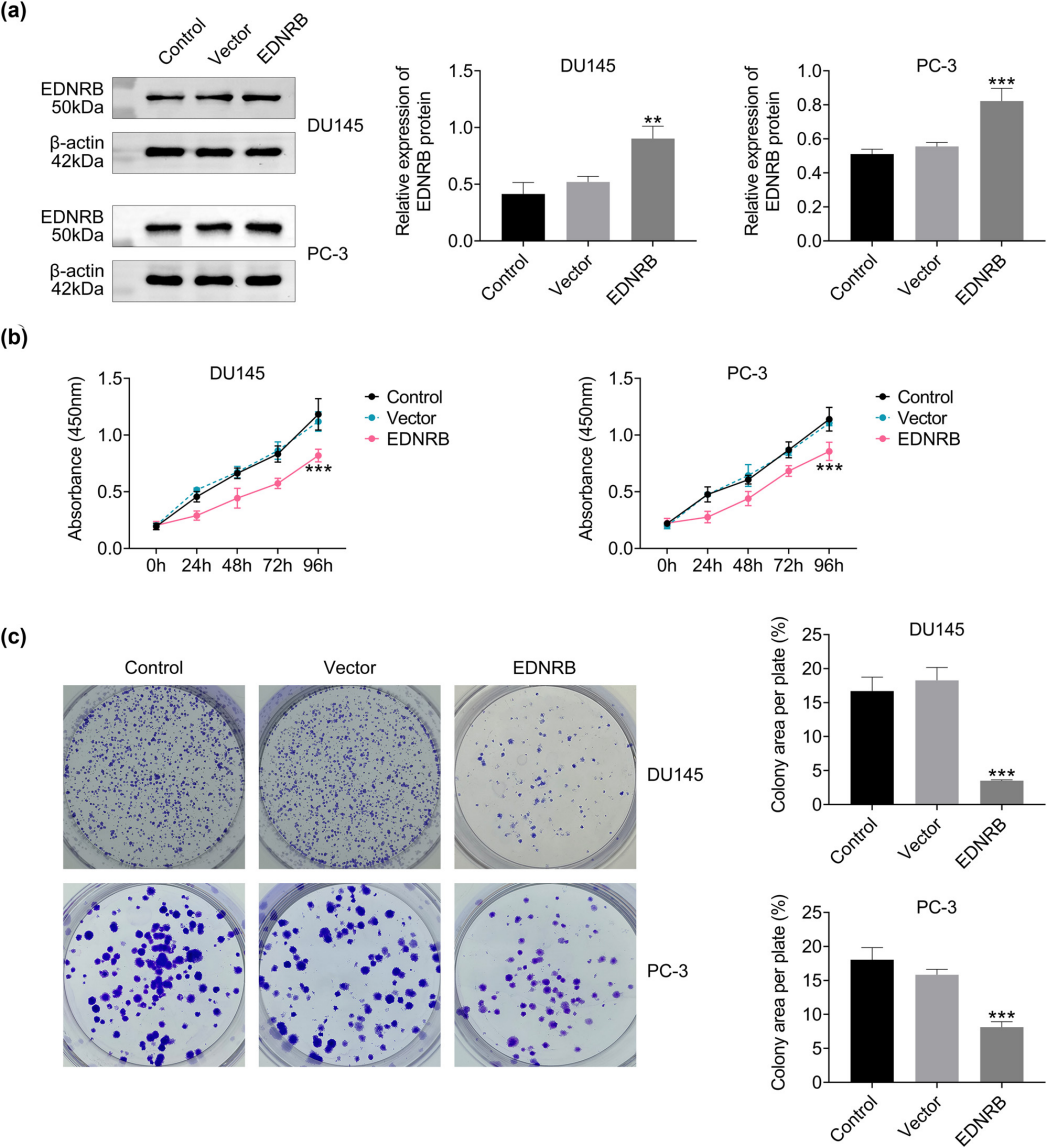
Effects of EDNRB1 overexpression on cell viability and colony formation ability of DU145 and PC-3 cells. (a) EDNRB1 protein expression level. (b) Cell viability changes at 24, 48, 72, and 96 h. (c) Cell clone proliferation. *N* = 3, **p < 0.01 and ***p < 0.001 vs the vector group.

The DU145 and PC-3 cells were selected to evaluate the impact of the EDNRB gene on the growth of PCa cells. EDNRB overexpression significantly increased EDNRB protein levels which suggests a high efficiency of overexpression, whereas vector transfection did not alter the expression level of EDNRB (Figure 5a). Cell viability was notably lower in the EDNRB overexpression group, with no such difference between the control and vector groups (Figure 5b). Colony formation experiments showed fewer colonies in the EDNRB overexpression group (Figure 5c). These results suggest that high EDNRB expression inhibits PCa cell growth.

### 3.4 EDNRB inhibits PCa cell migration and invasion

**Figure 6:**
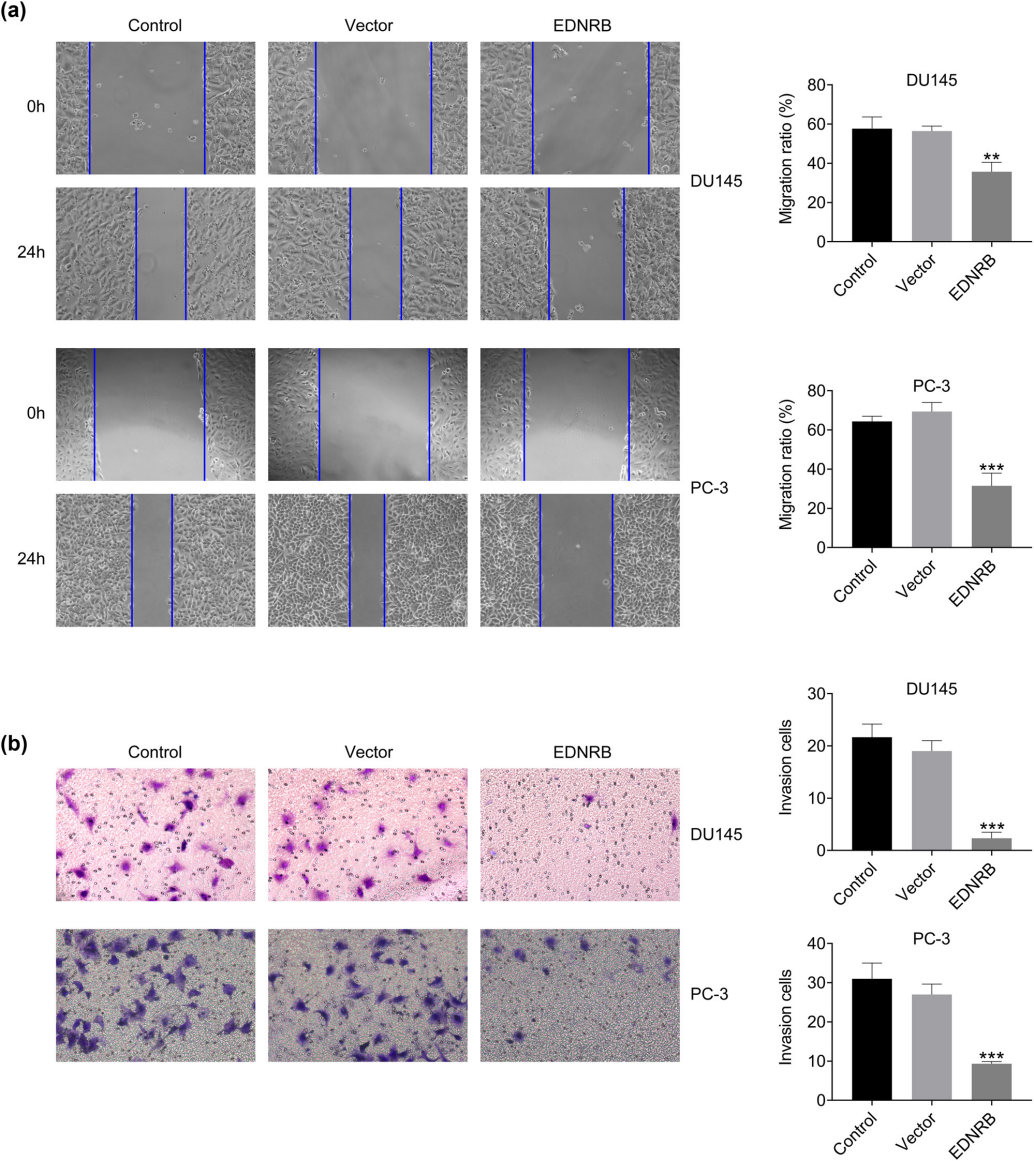
Effects of overexpression of EDNRB1 on the migration and invasion of DU145 and PC-3 cells. (a) The results of cell migration detected by wound healing assay within 24 h. (b) The situation of cell invasion detected by Transwell assay. *N* = 3, **p < 0.01 and ***p < 0.001 vs the vector group.

To evaluate the migration ability, we performed a scratch test on PCa cells. The results indicated that there was no noteworthy difference in the ratio of migrating cells between the vector group and the control group. However, when comparing the EDNRB overexpression group to the vector group, a significant decrease in the ratio of migrating cells was observed (Figure 6a). Similarly, the Transwell assay results consistently demonstrated that the overexpression of EDNRB significantly diminished the invasion ability of the cells (Figure 6b). These findings collectively suggest that the high expression of the EDNRB gene hampers both the migration and invasion capabilities of PCa cells.

### 3.5 EDNRB inhibits the growth of PCa cells by activating the cGMP-PKG pathway

**Figure 7:**
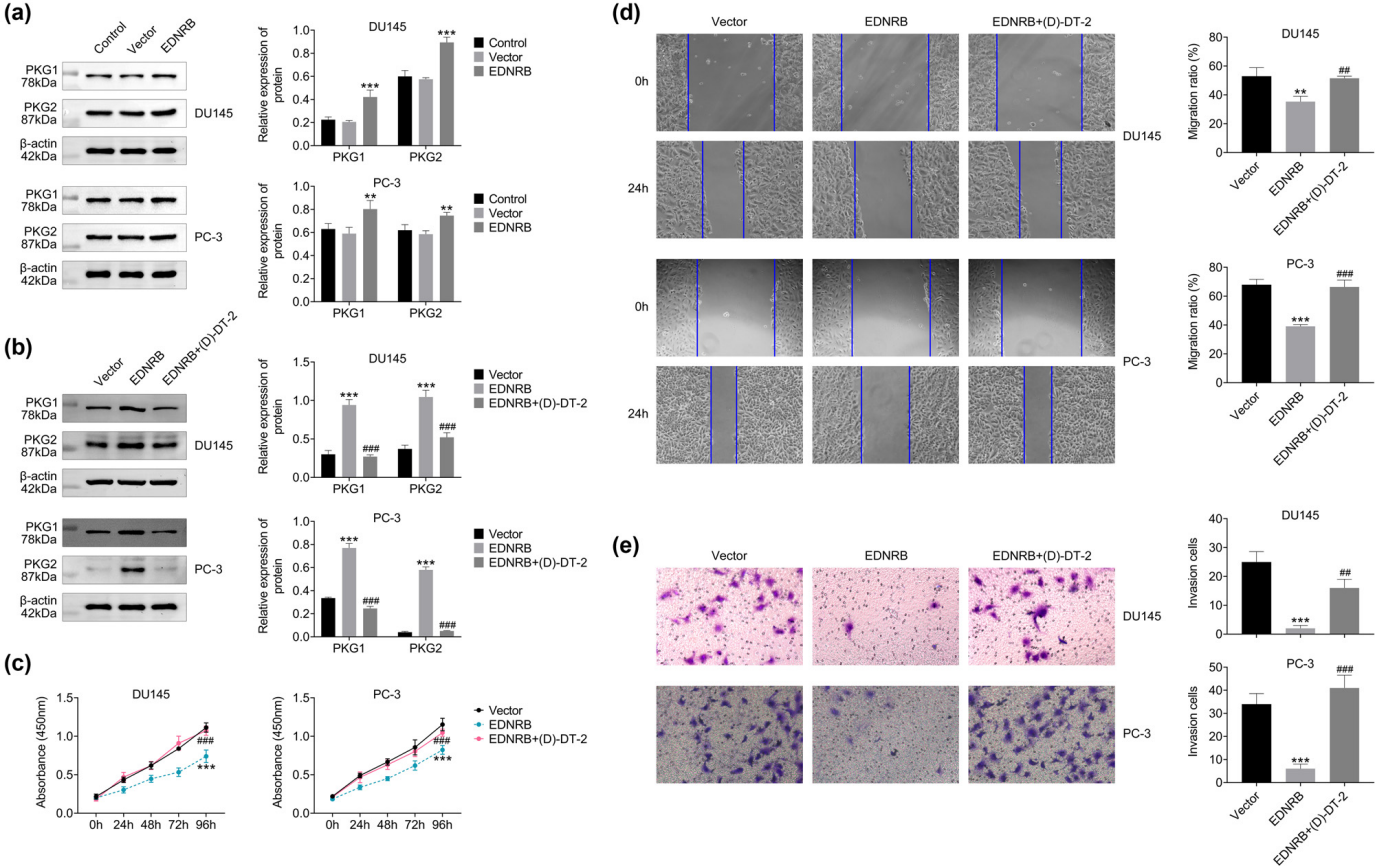
Regulatory effect of EDNRB1 overexpression on cGMP-PKG pathway. (a) Protein expression of PKG1 and PKG2. (b) Protein expression of PKG1 and PKG2 after introducing PKG inhibitor. (c) Cell viability after introducing PKG inhibitor changes over time. (d) The migration of cells within 24 h after the introduction of PKG inhibitors. (e) The invasion of cells after the introduction of PKG inhibitors. *N* = 3, **p < 0.01 and ***p < 0.001 vs the vector group; ^*##*^ *p* < 0.01 and ^*###*^ *p* < 0.001 vs the EDNRB1 group.

The effect of EDNRB on cGMP/PKG pathway was examined using WB. The results demonstrated no difference in PKG1 and PKG2 protein levels between the control and vector groups, but a significant increase was observed in the EDNRB overexpression group. This increase was suppressed by the PKG inhibitor (D)-DT-2, validating its effectiveness. EDNRB overexpression decreased PCa cell viability, but it increased with the introduction of inhibitors (Figure 7b).

Subsequently, we assessed the impact on the growth and colony formation ability of PCa cells. The results showed that, relative to the vector group, EDNRB overexpression decreased cell viability (Figure 7c) and colony formation (Figure 7d and e) but were restored by the inhibitors. These results suggested that EDNRB may inhibit PCa cell growth by activating the cGMP-PKG pathway.

### 3.6 EDNRB inhibits tumor growth *in vivo*

**Figure 8:**
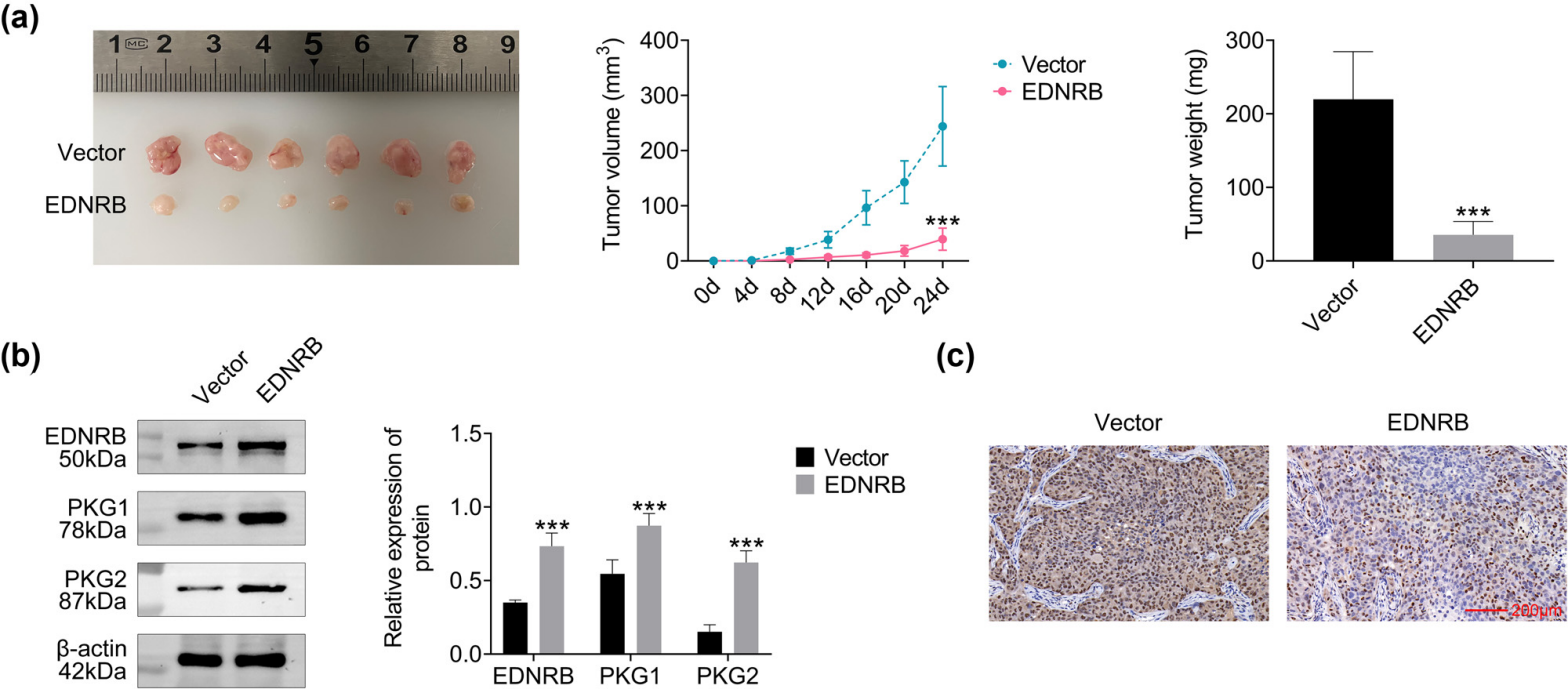
After constructing Balb/c nude mice, the effect of overexpression of EDNRB1 on PCa was detected. (a) Measurement of tumor size, volume, and mass every 4 days. (b) Protein expression of EDNRB1, PKG1, and PKG2 after overexpression of EDNRB1. (c) Ki-67 staining results of tumor sections shown by immunohistochemistry. *N* = 6, ***p < 0.001 vs the vector group.

A PCa model was established in Balb/c nude mice. and EDNRB overexpression resulted in significant differences in tumor size, volume, and mass compared to the control group (Figure 8a). WB analysis showed increased EDNRB, PKG1, and PKG2 protein levels in the EDNRB overexpression group (Figure 8b). Immunohistochemistry results displayed reduced ki-67 staining in this group, indicating less tumor growth (Figure 8c). These results validate the inhibitory effect of EDNRB on tumor growth *in vivo*.

## 4 Discussion

PCa is a highly prevalent malignant tumor in men, ranking second only to lung cancer in terms of incidence [10]. It poses a significant threat to men’s health, characterized by inconspicuous symptoms and often detected in advanced stages, thereby complicating disease management and control [11]. Despite the relatively high survival rate associated with early detection and treatment of PCa, many patients receive diagnoses only when they have already reached an advanced stage due to the subtle nature of early symptoms. Furthermore, PCa exhibits rapid progression upon relapse [12]. While several treatment options, such as new endocrine therapies, immunotherapy, and chemotherapy, are available for advanced PCa, none of these approaches offer long-term effectiveness in altering the disease’s ultimate outcome – death. Hence, it is of utmost importance to conduct comprehensive research and exploration in the field of PCa. This entails the identification of differentially expressed genes serving as biomarkers, the development of new therapeutic strategies and drug targets, and the enhancement of early diagnosis, disease prognosis, and patients’ quality of life.

Through an extensive analysis of public databases, our study successfully identified 414 differentially expressed genes associated with PCa, which potentially play crucial roles in its occurrence, development, and metastasis. Notably, we observed a significant downregulation of the EDNRB gene in PCa, suggesting its potential involvement in the initiation and progression of the disease. EDNRB encodes endothelin receptor B, a protein that exerts significant influence on various physiological processes like vasodilation and cardiomyocyte proliferation [13]. Altered expression of EDNRB in cancer profoundly impacts tumor cell proliferation, migration, and invasion. Inactivation of EDNRB has been identified as a pivotal factor in the development of certain cancer types, including melanoma and gastric cancer [6]. Numerous studies have established a close association between aberrant expression of the EDNRB gene and cancer occurrence, development, and prognosis, underscoring its importance in cancer biology. Previous research has elucidated the crucial inhibitory role of EDNRB in other cancer types [14]. Existing literature suggests an association between EDNRB downregulation and DNA methylation. For example, disruption of the endothelial axis in endometrial cancer was found by Nikola et al. The observed silencing of EDN3 activity may be primarily due to DNA methylation [15]. Aberrant epigenetic methylation alterations of EDNRB expression play an important role in the pathogenesis of hepatocellular carcinoma [16] and gastric cancer [17]. These studies have explored this relationship in different cancer types. We believe that future studies would benefit from a more comprehensive analysis that includes DNA methylation status. However, its specific function and molecular mechanisms in PCa remain elusive, necessitating further investigation.

Our study revealed that EDNRB may regulate the behavior of PCa cells by modulating the cGMP-PKG signaling pathway. This pathway plays a vital role in diverse physiological and pathological processes, encompassing cell proliferation, migration, and invasion. Its significance has been established in various cancer types [18]. For instance, studies have demonstrated that the activated cGMP-PKG pathway synergistically regulates the proliferation, migration, and invasion of PCa cells. In *Helicobacter pylori* infection, activation of the cGMP/PKG signaling pathway leads to the occurrence and development of gastric cancer, while blocking the PRTG/cGMP/PKG axis holds potential for gastric cancer treatment [19]. However, the specific function and regulatory mechanisms of the cGMP-PKG pathway in PCa remain unclear. Our study identified that high expression of the EDNRB gene inhibits the proliferation, migration, and invasion of PCa cells, offering valuable insights into the role of the cGMP-PKG pathway in PCa and the development of novel treatment strategies.

Undoubtedly, the novel finding of EDNRB’s low expression in PCa and its functional role in the cGMP-PKG pathway warrants further investigation. First, our observations regarding the low expression of EDNRB in PCa tissues and cell lines require validation in larger patient sample cohorts. Moreover, variations in gene expression levels may exist due to differences in sample sources, potentially impacting our results. Second, although our study revealed that high expression of the EDNRB gene activates the cGMP-PKG pathway, the intricate molecular mechanisms underlying the precise regulation of this pathway by EDNRB remain unclear. Further research is necessary to delve deeper into these mechanisms, including exploring potential intermediary molecules involved in the regulation of the cGMP-PKG pathway by EDNRB [20]. Our current study did not assess the DNA methylation status of the EDNRB gene, which may be a significant factor in its downregulation in PCa. The potential role of epigenetic modifications in EDNRB expression represents an important aspect of gene regulation that warrants further exploration. Future research efforts will be directed to include an analysis of the DNA methylation status of EDNRB to provide a more comprehensive understanding of its involvement in PCa. Third, while our study provides valuable insights into the role of EDNRB overexpression in PCa, we recognize the absence of knockdown or knockout controls as a limitation. The importance of such experiments cannot be overstated as they would offer a more complete understanding of EDNRB’s role by observing the effects of its reduced expression or complete absence. Future studies will aim to include these control experiments to validate the findings presented herein. Our study used (D)-DT-2 to inhibit the PKG pathway; however, the broad specificity of (D)-DT-2 might affect other molecular pathways. Direct genetic knockout of key genes within the PKG pathway would provide stronger evidence for the specificity of the pathway’s role in the observed effects of EDNRB on PCa cells. We aim to undertake these more targeted genetic manipulation experiments in future research to solidify our conclusions. Additionally, while our *in vitro* experiments demonstrated the inhibitory effects of high EDNRB expression on the growth and migration of PCa cells, it is crucial to acknowledge the substantial disparities between the *in vitro* and *in vivo* environments [21]. The *in vivo* setting is more complex, involving numerous interactions and signaling pathways. Therefore, although we validated the inhibitory effect of EDNRB in a nude mouse model, further verification using additional animal models and clinical samples is imperative for a comprehensive understanding of EDNRB’s function and role *in vivo*. Additionally, our research did not encompass the impact of EDNRB on the prognosis of PCa patients, which constitutes an important avenue for future investigation.

In summary, our study sheds light on the significant role of EDNRB in PCa. Nonetheless, certain limitations exist, necessitating further research and exploration. This includes elucidating the detailed mechanism of action of EDNRB in PCa, verifying its tumor suppressor effect in larger clinical sample sizes and diverse animal models, as well as investigating its impact on the prognosis of PCa. We anticipate that through more extensive research, a comprehensive understanding of EDNRB’s role in PCa can be achieved, ultimately translating these findings into clinical applications and providing novel strategies for the treatment of this disease.

## 5 Conclusion

Our study demonstrates the significant involvement of EDNRB in PCa, wherein it exerts inhibitory effects on tumor cell growth, migration, and invasion through the activation of the cGMP–PKG pathway. These findings not only enhance our comprehension of the underlying mechanisms driving PCa pathogenesis, but also identify potential targets for the development of novel treatment strategies, thus offering promising prospects and opportunities.
